# “They think you’re trying to get the drug”: Qualitative investigation of chronic pain patients’ health care experiences during the opioid overdose epidemic in Canada

**DOI:** 10.1080/24740527.2021.1881886

**Published:** 2021-04-15

**Authors:** Lise Dassieu, Angela Heino, Élise Develay, Jean-Luc Kaboré, M. Gabrielle Pagé, Gregg Moor, Maria Hudspith, Manon Choinière

**Affiliations:** a Carrefour de l'innovation et de l'évaluation en santé, Research Center of the Centre Hospitalier de l’Université de Montréal (CRCHUM), Montreal, Quebec, Canada; b Department of Biomedical Sciences, Faculty of Medicine, Université de Montréal, Montréal, Quebec, Canada; c Pain BC Society, Vancouver, British Columbia, Canada; d Department of Pharmacology and Physiology, Faculty of Medicine, Université de Montréal, Montreal, Quebec, Canada; e Department of Anesthesiology and Pain Medicine, Faculty of Medicine, Université de Montréal, Montreal, Quebec, Canada

**Keywords:** chronic pain, opioids, stigma, discrimination, health inequalities, qualitative research

## Abstract

**Background**: The opioid overdose epidemic has led health care providers to increased vigilance for opioid-related risks in the treatment of chronic non-cancer pain (CNCP). Media have conveyed stigmatizing representations of opioid analgesics.

**Aims:** This study aimed to understand how the opioid overdose epidemic has impacted health care experiences among people living with CNCP in two Canadian provinces (British Columbia, Quebec).

**Methods:** This qualitative study proceeded through 22 semi-structured interviews conducted in 2019. Participants were recruited from a cross-sectional survey examining the effects of the opioid overdose epidemic on individuals with CNCP. We collected in-depth narratives that we analyzed using a thematic framework. The sample included 12 women and 10 men aged 20 to 70 years, with 11 from each province.

**Results:** Several participants described increased difficulty in accessing medical services for pain since the onset of the opioid overdose epidemic. They reported that some physicians urged them to taper opioids regardless of their pain severity and functional limitations. Some participants reported facing discrimination and care denials as they were labeled “drug-seeking,” especially in hospital. Depending on their educational resources, they were unequally able to counter providers’ stigmatizing behaviors. However, participants described empathetic relationships with providers with whom they had a long-term relationship. Some participants drew distinctions between themselves and the stigmatized status of “addict” in ways that reinforced stigma toward people who are dependent on opioids.

**Conclusions:** Health policies and provider education programs aimed at reducing opioid-related stigma are needed to counter detrimental consequences of the opioid overdose epidemic for people living with CNCP.

## RÉSUMÉ

**Contexte**: L’épidémie de surdose d’opioïdes a conduit les prestataires de soins de santé à une vigilance accrue des risques liés aux opioïdes dans le traitement de la douleur chronique non cancéreuse (DCNC). Les médias ont véhiculé des représentations stigmatisantes des analgésiques opioïdes.

**Objectifs**: Cette étude visait à comprendre comment l’épidémie de surdose d’opioïdes a eu un impact sur les expériences avec les soins de santé des personnes vivant avec la DCNC dans deux provinces canadiennes (Colombie-Britannique, Québec).

**Méthodes**: Cette étude qualitative a été réalisée au moyen de 22 entretiens semi-structurés menés en 2019. Les participants ont été recrutés à partir d’une enquête transversale examinant les effets de l’épidémie de surdose d’opioïdes sur les personnes atteintes de DCNC. Nous avons recueilli des récits approfondis que nous avons analysés en utilisant un cadre thématique. L’échantillon comprenait 12 femmes et 10 hommes âgés de 20 à 70 ans, parmi lesquels 11 provenaient de chacune des deux provinces.

**Résultats**: Plusieurs participants ont décrit une difficulté accrue à avoir accès aux services médicaux pour la douleur depuis le début de l’épidémie de surdose d’opioïdes. Ils ont déclaré que certains médecins les avaient incités à réduire les opioïdes, quelle que soit la gravité de leur douleur et leurs limitations fonctionnelles. Certains participants ont déclaré être confrontés à de la discrimination et au refus de soins car ils ont été qualifiés de « chercheurs de drogue », en particulier à l’hôpital. En fonction de leurs ressources éducatives, ils ont été inégalement capables de contrer les comportements stigmatisants des prestataires. Cependant, les participants ont décrit des relations d’empathie avec les prestataires avec lesquels ils avaient une relation à long terme. Certains participants ont établi des distinctions entre eux-mêmes et le statut stigmatisé de « toxicomane » d’une manière qui renforce la stigmatisation des personnes dépendantes aux opioïdes.

**Conclusions**: Les politiques de santé et les programmes d’éducation des prestataires visant à réduire la dépendance aux opioïdes et la stigmatisation liée aux opioïdes est nécessaire afin de contrer les conséquences néfastes de l’épidémie de surdose d’opioïdes pour les personnes vivant avec la DCNC.

## Introduction

Growing evidence on the detrimental effects of long-term opioid therapy^1^^,2^ and a recent drastic increase in opioid-related mortality in North America^3,4^ have led health regulators to enact new opioid prescription guidelines addressing the “opioid overdose epidemic.” These guidelines typically promote opioid tapering or cessation and advise against long-term opioid prescription to prevent risks of dependence and serious adverse events such as overdoses.^5,6^ This new era of “opioid pharmacovigilance”^7^ is catalyzing major changes in the medical management of people living with chronic non-cancer pain (CNCP), given that prescription opioids are now more difficult to obtain overall.^8–11^ Some recent studies suggested that patients with CNCP treated with opioids may experience new barriers in accessing their usual opioid medication due to prescribers’ increased concerns following recent opioid regulations.^8,9,12,13^

In addition, the media coverage of the opioid overdose epidemic can lead to further stigmatizing people living with CNCP.^8,14,15^ Even though the overdose deaths mainly involve substances coming from the illicit market such as counterfeit fentanyl^16–19^ or polyconsumption of opioid and nonopioid substances,^20^ the media often created confusion with opioids prescribed for CNCP.^14^ Results of an online survey conducted in 2018 by our research team revealed that the media coverage of the opioid overdose epidemic was perceived by people living with CNCP as very detrimental whether they took opioids or not.^21^ Indeed, another recent Canadian study showed that newspapers covering the opioid overdose epidemic usually depicted patients with CNCP as either becoming dependent on opioids against their will or intentionally seeking drugs.^14^ In part due to this media coverage, the social stigma that has long been associated with illicit substance use^22,23^ may now extend to people suffering from CNCP who use prescribed opioids.^8,15,23,24^

Both clinicians’ increased pharmacovigilance and opioid stigma conveyed in the media may raise concerns for patients’ access to health care services for CNCP. This study aims to better understand the concrete situations in which the opioid overdose epidemic can affect the health care experience and interactions with providers of people living with CNCP. How and in what ways does opioid-related stigma create barriers to care for people living with CNCP?

This study is grounded in Goffman’s interactionist conceptualization of stigma as a hidden sign of a socially devalued identity^25^ and Corrigan and Nieweglowski’s analysis of how stigmatizing stereotypes can lead to situations of discrimination and to label avoidance behaviors.^26^ The consequences of stigma on structural health inequities, as conceptualized by Link and Phelan^27^ and Hatzenbuehler et al.^28^ were also considered in this study. The theoretical framework of stigma enabled us to analyze how interactions with health care providers^29^ may convey negative stereotypes regarding opioids and lead to discriminatory practices against people living with CNCP treated with these medications.

## Methods

### Design

This qualitative study is part of a sequential explanatory^30^ mixed methods project assessing the impact of the opioid overdose epidemic on people living with CNCP (for quantitative data, see Kaboré et al.).^21^ This study was held in two Canadian provinces chosen because of their contrasting situation regarding the opioid overdose epidemic: Quebec, which ranks among the provinces with the lowest rates of opioid-related overdoses, and British Columbia, the most affected province.^4^ The study’s qualitative component intended to understand how the opioid overdose epidemic impacted participants’ daily living conditions, namely, their health care experiences and social relationships. This article focuses on health care experiences, and a forthcoming article will examine social and personal impacts. We proceeded through one-on-one semi-structured phone interviews among a subsample of respondents to our previous online quantitative survey.

The study was approved by the Ethics Committee of the Centre Hospitalier de l’Université de Montréal (No. 17.177-MP-02-2018-7286).

### Sampling and Recruitment

A purposive sample of 22 participants was recruited between March and July 2019. The final number of participants was guided by data saturation; that is, recruitment stopped when information provided in new interviews became redundant with information from previous interviews.^31,32^

**Figure 1.**
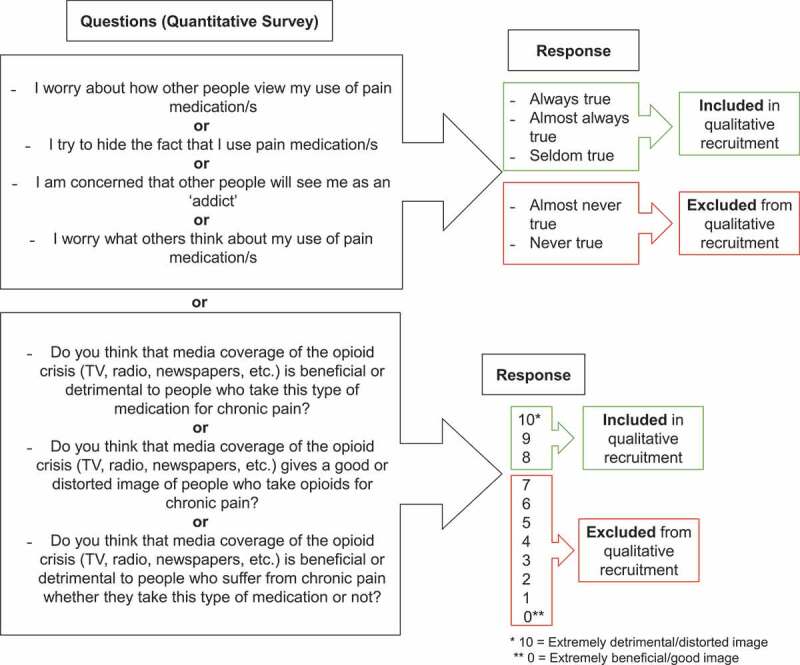
Study eligibility criteria

Only the respondents in the quantitative survey who allowed the research team to communicate with them for further studies were considered for participation. The main inclusion criterion was having reported perceived stigma; that is, concerns about other peoples’ judgments regarding opioid use and/or perceived negative impacts of the opioid overdose epidemic’s media coverage in the initial survey (see Figure 1 for inclusion criteria). We used this criterion because we believed that further understanding the experience of participants reporting perceived stigma would improve knowledge of the impacts of the opioid overdose epidemic on people with CNCP.

To encompass a wide range of experiences, we added several diversification criteria: We ensured recruitment of both women and men, people of various ages, and individuals living in Quebec and British Columbia. A few participants who did not use opioid analgesics were included to examine differences in the impact of the opioid overdose epidemic in this population. Participants were required to be 18 years of age or older and able to give informed consent electronically.

A total of 108 eligible participants were sent an e-mail presenting the study and offering them the opportunity to take part in a qualitative phone interview. Among those who responded positively (*n* = 40, 37%), we chose a subsample matching the study diversification criteria (province, gender, age, opioid use). These participants were contacted on the phone or by e-mail by the first author (LD) or the second author (AH) to schedule an appointment for the interview.

The final sample of 22 participants included 12 women and 10 men, with 11 from each province; their ages ranged from 20 to 70 years. Fifteen of them used a prescribed opioid treatment for CNCP and 3 had been prescribed opioids in the past but had stopped at the time of the interview. The other 4 participants had never been prescribed opioids for CNCP. Minimum pain duration was 2 years and maximum was 39 years. Detailed characteristics are provided in Table 1.

**Table 1.** Participants’ characteristics

Characteristic	*N*
Total number of participants	22
**Age**	
20–29	4
30–39	3
40–49	7
50–59	4
60–69	3
≥ 70	1
**Gender**	
Female	12
Male	10
**Province of residence**	
British Columbia	11
Quebec	11
**Prescribed opioid treatment for CNCP**	
Currently treated with opioids	15
Treated with opioids in the past	3
Have not been treated with opioids for CNCP	4
**Pain duration**	
1–5 years	3
6–10 years	2
11–15 years	3
16–20 years	6
21–25 years	4
>25 years	4
**Provider(s) treating CNCP**	
Multidisciplinary pain treatment clinic	4
Family physician	13
Specialist	5
No follow-up for CNCP	1
On waitlist for multidisciplinary pain treatment clinic	2
**Education level**	
Secondary	2
Technical/college	10
University	10

### Data Collection

Interviews in Quebec were conducted by the first author (LD), a francophone health sociologist. Interviews in British Columbia were conducted by the second author (AH), an anglophone research assistant trained in nursing sciences. Both had high-level training and long experience in qualitative research among people in pain and vulnerable populations. For this article, French-to-English translation of the quotes from Quebec participants was conducted and checked by four bilingual members of the research team.

Interviews lasted 65 min on average (40–130 min). Participants were informed that they could interrupt the interview at any time if they wished to take a break (which happened only once). After interviews, participants were sent monetary compensation of CA$50.00 for their time. All interviews were audio-recorded and transcribed verbatim with de-identification of all names, places, and dates. In this article, participants’ names were replaced with pseudonyms.

The semi-structured interview guide included open-ended questions inviting participants to produce in-depth narratives of their current relationships with health care providers (physicians, nurses, pharmacists) and with their family, friends, and coworkers. Participants were invited to describe how the opioid overdose epidemic had impacted these domains of their life. They were also asked to share their thoughts regarding recent opioid policy changes and the media coverage of the opioid overdose epidemic. The interview guide also included questions about the characteristics of their pain and their treatment history.

### Data Analysis

Interviews were analyzed using a thematic analysis framework, as defined by Miles et al.^32^ This framework identifies common patterns of meaning (themes) through a comparison of participants’ narratives. Thematic analysis was particularly suitable to this project because this method offered a balance between the focus on our initial research questions and an inductive approach to data allowing production of emerging meaning.

Data collection and analysis occurred simultaneously and continued until we reached data saturation.^31,33^ The first author (LD) analyzed all of the interviews. The first six interviews (approximately 25%) were analyzed by the first and third authors (LD, ED) to build a preliminary and evolving codebook.^34^ Involvement of two analysts and regular team discussion enriched the analysis and reinforced validity of data interpretation.^35,36^ We used NVivo software^76^ to organize interview excerpts and group them into codes. Open coding of each interview enabled us to produce meaning from interview excerpts. Then, we examined relationships between codes through axial coding, which enabled us to produce more general and conceptual themes.^33^ We used an iterative coding procedure: We first interpreted data based on our research questions and the topics appearing in the interview guide. During the analysis process, we wrote memos to describe variations in participants’ narratives and progressively build emerging interpretations.^33^ The themes presented in this article do not exactly reflect the topics initially present in the interview guide; rather, the organization of themes and their content result from the meaning produced through coding procedures, memo writing, and interpretation of participants’ narratives. In this article, we focus on five themes related to the impacts of the opioid overdose epidemic on participants’ health care experiences. However, during the analysis process, we developed more themes that will be described in another publication.

## Results

This section describes participants’ health care experiences and interactions with providers during the opioid overdose epidemic. We identified five main themes from interviews: (1) opioid tapering as a standardized response from providers; (2) experiences of stigma and discrimination in health care; (3) positive long-term, trusting relationships with health care providers; (4) structural inequities in countering providers’ stigmatizing behaviors; and (5) distancing from the stigmatized status of “addict.”

### Opioid Tapering as a Standardized Response

Some participants reported that, as a result of recent opioid prescription guidelines, physicians suddenly urged them to quickly taper opioids regardless of their medical history, pain severity, and functional limitations. In most cases, participants felt that they were not listened to. They deplored that physicians’ standardized responses of tapering or ceasing opioids did not consider their personal priorities and needs for functioning.

Some participants explained that they were willing to taper opioids progressively, but they would do it at their own pace to prevent negative consequences on their pain and stress. They typically felt that physicians took those decisions without considering their opinions and capabilities:

I noticed when I was in pain that I was binge [eating] more [NB: this participant lives with an eating disorder]. So, I went to my doctor and said, “I’m not sure if [ceasing my opioid treatment] is the right direction to go,” and he said, “Well, I’m not willing to keep prescribing you morphine.” I said, “Well, that wasn’t the discussion before we started. You asked me if I would go off of it and I said, ‘Of course, if you could find something else that works.’” He wasn’t willing to prescribe anything else. (Samuel, 40–49, British Columbia, treated with opioids)

My doctor, since I opened the door to reducing narcotics … I went from 30 milligrams of morphine to 15 milligrams and then I was down to using about four tablets of morphine a day. It’s not much anymore. Then he wanted to wean me off super-fast. I was like: “No, it doesn’t work, Doctor, stop!” (Sylvie, 40–49, Quebec, treated with opioids)

Some other participants reported that they had already tried and failed many treatments before they had been prescribed opioids as a last resort. They deplored that providers typically did not consider this in their decision:

Initially it was okay, and then when the changes came into place with the College [of physicians], it was very difficult to get a prescription from my GP [general practitioner], even with a letter from my pain specialist telling my GP he needed to provide a hydromorphone prescription. Because the gabapentin—everything else—was not working and I needed the hydromorphone for pain management. (Irene, 40–49, British Columbia, treated with opioids)

According to participants, health care providers who had little knowledge of participants’ pain history were more likely to impose opioid tapering or quick withdrawal or switch to a nonopioid treatment. Several participants reported that unsolicited reconsideration of their treatment usually happened when meeting a new physician or new pharmacist, a resident, or a locum.

The fear that some doctors have of me taking narcotics. It makes them very afraid. Quite often, some residents met me in the hospital and wanted me to stop taking these drugs. They offered me a withdrawal plan, whereas I had never asked for it; it was not their role to do it because they didn’t manage the condition for which I take this medication. It looks like, and probably for good reason, the opioid crisis is creating hypervigilance among health professionals and there’s a lot of pressure to give up these treatments. I find myself very mistreated, while the suffering we are experiencing is real. Quite often, we use narcotics for a physical pathology which unfortunately is not curable, so it’s perhaps our only way to function and remain active in society. I think it’s important. At [my age], I don’t want to be considered disabled. (Paul, 30–39, Quebec, treated with opioids)

In several cases, participants thought that physicians’ “fear” of prescribing opioids resulted from the media coverage of the overdose epidemic. Some participants felt that this situation was unfair, especially those who considered their opioid consumption to be moderate and self-controlled:

[My medical specialist] preferred to prescribe me Hydromorph Contin, which is long-acting, rather than Dilaudid every four hours, which I used to take before. But I know there are days when I don’t take any. [The Dilaudid] gives me a dose when I need it. The fear he had when telling me this, that he liked long action better than short action, was that he said that short action could create addiction or that I was going to ask him for more because at some point I was going to get used to this dose. I am not. It’s been 12 years anyway. I also feel that with the media coverage of opioids, doctors are … instead of being informed they are frightened. (Allison, 50–59, Quebec, treated with opioids)

### Experiences of Stigma and Discrimination in Health Care: When People in Pain are Labeled “Drug-Seeking”

Several participants reported that they faced situations of stigma and discrimination related to their opioid treatment, which could take two distinct forms: health care denials and stigmatizing behaviors. Both occurred when providers labeled participants as “drug-seeking.” According to participants, these problems were intensified during hospitalizations and visits to the emergency room, which increased participants’ feelings of vulnerability when using these services.

In many cases, participants reported that their access to pain relief in hospital was determined by providers’ initial perceptions of the reason for their visit. Due to opioid treatment, several participants reported that they were hardly considered as legitimate patients.

I think that’s the effects of this opioid crisis that we’re going through now, I think it had a bearing on the way I was treated in the hospital when I went to the ER [emergency room] at times. Because, of course, they think you’re trying to get the drug, or you’re addicted to it, and things like that. It was difficult at times, depending on the doctor I would get. (Patrick, 60–69, British Columbia, treated with opioids)

When participants would experience a one-off problem with their opioid prescription (e.g., losing tablets or coming one day earlier than prescribed), they reported that they were sometimes labeled “addicts” and denied CNCP treatment due to allegations of noncompliance or drug-seeking. Several of them felt trapped and powerless to prevent these situations:

[When I moved to town], I remember putting my opioid pills in an extremely safe place. But, when we got down here, I couldn’t remember at all. I started going through things and trying not to panic, and then I started trying to find a doctor who would help me out, and that’s when the nightmare really began. It’s hard to explain. The things they said, wow! “I bet you never misplaced your blood pressure medication,” which I wasn’t even on, but just accusatory statements. And it would be no point trying to tell them that with fibromyalgia this would be called brain fog, and I couldn’t find my purse once for a couple of days because I put it in the fridge. You do things and they’re gone from your mind sometimes. Each doctor treated me really horribly. I was suddenly a drug-seeking addict. I went from patient to [addict], like “bang,” just like that. […] So, what was just something that could happen to anybody during a hectic move like that, a million things to consider, suddenly turned me into this drug-seeking addict in the eyes of every doctor I went to see. (Bettina, 70–79, British Columbia, treated with opioids)

When the stigmatizing label of “addicts” came to be noted in medical records, it could lead to extremely detrimental consequences for participants’ health care trajectories, as happened to a participant who reported that he lost his family physician after facing discrimination in the ER:

I was in a lot of pain in my abdomen, this was postsurgery, and went into the [hospital]. It was a weekend, and the young doctor I saw was totally overworked, and when he couldn’t find anything that was happy with him, he basically just said, “Look, you’re here looking for drugs. I don’t need people like you in my ER and I’d like you to leave.” Not only that, he ended up making a complaint to my family doctor, and my family doctor who was familiar with me but obviously didn’t know about the interaction at the hospital, now all of a sudden my family doctor basically said, “Look, you’re a drug addict, you’re a drug seeker, and I just don’t want to be involved in this process with you.” So, I lost a doctor. (George, 50–59, British Columbia, treated with opioids)

In addition to explicit care denials, several participants reported more diffuse forms of discrimination: situations in which they felt negatively judged or roughly treated by providers. Several of them used words such as “treated like a drug addict” to describe these inhumane and stigmatizing interactions:

What I find difficult in taking opiates is the way health professionals look at you. I feel that in pharmacies … due to the current shortage of pharmacists, I often see locum pharmacists who move in the network, they do not know their patients, and I find that they judge very easily or they will treat you like a junkie instead of a sick person. (Paul, 30–39, Quebec, treated with opioids)

Fear of discrimination when going to the ER brought some participants to waive their pain management regimen in attempts to gain providers’ trust. To prevent negative judgment, some participants explained that they preferred not asking for opioids:

When I go to the ER—and truly, you might find this interesting—but the second you tell an ER doctor that you don’t want narcotics, you get treated differently. So, I really felt that they were really responsive to that, and they were saying, “Okay, this girl isn’t looking for what other people might be looking for.” But I also probably injured myself in doing that, because I denied myself relief, probably. So, perception certainly has played a very large role in how I’ve managed it and being concerned about those judgments. (Jane, 30–39, British Columbia, treated with opioids in the past)

### Long-term Trusting Patient/provider Relationships Protect from Stigma in Healthcare Environments

Most of the difficulties reported by participants occurred with providers who were unfamiliar with them. When a long-term trusting relationship was established, there were fewer risks of being discriminated against. However, it may be challenging to build such relationships with new or unknown providers. One participant treated with opioids described the contrast between her positive relationship with her former family physician and challenges in finding a new family physician after moving to another town:

My former physician, who had got me started on opioids, he had been my doctor for about eight years, he knew me and the kind of work I did and my pain history. He was a caring doctor. He didn’t push anything on me. He just knew my situation and wanted to help me be able to function again at somewhat near the level I did before. It worked well and he was delighted to see the changes in me and how healthy I was able to get myself again. That was part of the problem after I moved down here, because I was slim and strong, tanned, I felt really healthy. I didn’t look like somebody who needed heavy-duty opioids. So that worked against me also. [Physicians] just wanted me out of their office. They didn’t believe I was a chronic pain patient. (Bettina, 70–79, British Columbia, treated with opioids)

According to many participants, trusting relationships with health care providers were of the utmost importance in accessing adequate pain management. Some participants considered themselves fortunate to have a family physician during the opioid overdose epidemic:

P: Not a lot of people have family doctors. That’s key, really. To me, if you don’t have a family doctor that you can build a relationship with, pain management is next to impossible. Half the problems I hear about are mostly people fighting for it, because they don’t have anyone they can build a trusting relationship with to do that type of long-term care for it.

R: That continuity of care is so important, isn’t it?

P: Yeah, totally, especially when even in the professional field right now, I had a couple of specialists that just made judgment calls on other diagnosis with me just based on “oh,” because you’re on opiates. (Chris, 40–49, British Columbia, treated with opioids)

Several participants described positive long-term relationships with family physicians, specialists, pharmacists, or nurses. Such relationships were particularly reassuring because providers were committed to participants’ follow-up and understood their medical history, needs, and demands:

Luckily, I have good doctors. I see my neurologist, because I have Parkinson’s also. So, I see my neurologist for that. I have a pain doctor, Dr. A., I have Dr. B. who works with Dr. A., he’s a psychiatrist for pain. I saw Dr. C. at one point when I was trying Suboxone out, […] Dr. C. was the person who I went through for that. And, of course, my GP, Dr. D. All five of those doctors totally understand where I’m coming from and how I’m dealing with the pain, and they’re very respectful. They are always looking for answers, all five of them. (Patrick, 60–69, British Columbia, treated with opioids)

She really looked for [a diagnosis], I didn’t feel like she just said, “Okay, I gave you medication, now go, that’s what you wanted!” I really liked that, because she was asking me about which side effects I could tolerate and which I would not tolerate. So, I was involved in deciding which medication I was ready to try or not depending on what was the worst for me. (Geneviève, 20–29, Quebec, not treated with opioids)

Though these positive relationships were described by several participants using opioids, those who were not treated with opioids typically reported fewer negative interactions with health care providers.

### Countering Providers’ Stigmatizing Behaviors: the Burden of Structural Inequities

When it came to obtaining pain relief, many participants treated with opioids stated how challenging it was to be listened to and believed as a patient with chronic pain. To avoid opioid-related stigma and discrimination, most participants reported that they had to deploy communication skills and resources to advocate for their case and convince providers of the legitimacy of their medication requests. Depending on their ease with oral communication and medical literacy, they were unequally able to acquire and mobilize advocacy skills.

One participant mentioned the “social skills” he had to mobilize to defend his case in the ER. He reported that he had the opportunity to learn those skills at work and was concerned that other patients would not be able to act like he did:

The ER doctor, his first initial thing is the “no.” So, then again, I’m hoping that someone is just going to listen to me. Eventually he did listen, but it was a really degrading experience. At the same time, it made me really sad because I have good social skills. I was in the nightclub industry for a long time, I know how to talk to people. And it made me sad because I thought of all the people that are out there, that were maybe going through something similar, that didn’t have social skills, or didn’t know how to act, or maybe were even sicker than me, and didn’t even know how to handle it, that would maybe just go home and suffer. (Samuel, 40–49, British Columbia, treated with opioids)

Indeed, for many participants, convincing providers had become a work per se in the management of their pain. Though they were not entirely protected from discrimination, participants who could access and understand scientific information about their condition reported that they had more chances to counter the “addict” stigma and eventually obtain their treatment using self-advocacy.

I think I was informed, and I think I could advocate myself, and I think I spoke the medical language, which I think really played to my benefit. I could describe things in a language that they could understand and I knew the physiology piece, and I had really done my homework and I think I really tried, so I think they just responded well to me in that sense. (Jane, 30–39, British Columbia, treated with opioids in the past)

One participant also mentioned his privileged social status and sociodemographic proximity with his physician:

My family physician now, I think he definitely does listen to me. But I also think he listens a little bit more because I’m a white male, middle class, in the health studies, so I feel like I have a lot of privilege, whereas I know other people who are in chronic pain don’t have that same kind of privilege and aren’t listened to as much. (Trevor, 20–29, British Columbia, treated with opioids)

Conversely, participants with less scientific/medical literacy and knowledge of the health care system were more at risk of failing to convince providers that they were legitimate patients with chronic pain. They lacked resources to advocate for their case and get the stigmatizing label of “addict” removed from their medical files. Stigma and discrimination could therefore lead to more negative long-term consequences in their access to pain management. For example, one participant reported that she was unable to identify the causes of the discriminations she faced until her family physician told her that providers may have prejudices against her treatment:

My family doctor made me realize that. He told me, “Christine, do you know that when you are in the hospital, the staff doesn’t know that opioids are for your chronic back pain?” I said, “Do you think so?” He said, “Yes, when I was on duty the other day, I even heard them say, ‘Ah, methadone, they are in withdrawal, don’t give anything.’” (Christine, 40–49, Quebec, treated with opioids)

The case of another participant is emblematic of the dramatic consequences of resource inequities for countering stigma. She explained that after having failed to convince several physicians to refill her opioid prescription, she felt powerless to seek medical help. After a couple of weeks of suffering from opioid withdrawal and intense pain, the only solution she could find was to enter an opioid dependence treatment program, thus officially endorsing the stigmatized status of “addict:”

I ended up having to go to the ER to try to get just a couple days’ worth. And [the physician] looked at my chart and saw when I was last prescribed the medication, and I realized if I said, “Oh, I lost it” that would just sound like a bunch of BS. And I started to realize that the ERs were getting swamped with people saying they lost, or they had their medication stolen, on and on. All of a sudden I was in that group of people according to them. She gave me two days’ worth, which I was really grateful for, because it gave me a bit of time to try and figure out what to do. So, there I was, basically I was totally on my own down here and it was terrifying. [Withdrawal symptoms] went on for three weeks, at which point I felt like I was hitting the breaking point because I was in terrible shape. I realized I had to do something or my whole life is, you know, I’m going to end up homeless or die on the street. So, I checked out. I realized since they considered me an addict anyway, I might be able to get on a methadone program. (Bettina, 70–79, British Columbia, treated with opioids)

### “The Bad Apples”: When People in Pain Distance from the Stigmatized Status of “Addict”

Many participants’ narratives were punctuated with attempts to differentiate themselves from those they called “addicts,” building a strict boundary between “us” and “them.” Indeed, several participants thought that they were unfairly affected by the overdose epidemic’s consequences. According to them, patients with pain should not be involved in controversies regarding opioids.

I think, though, that the majority of the opioid crisis comes from illicit medication that are cut into preexisting drugs that are on the streets, and there’s a lack of knowledge. I think there definitely needs to be something done, but I don’t think the drugs they get on the street are coming from a medical provider. They’re coming from China or they’re coming from online, or they’re coming from somewhere else, and that’s something they need to look into instead of punishing the people that require them for daily life. (Hannah, 30–39, British Columbia, treated with opioids)

Some participants felt outraged that providers could equate them with “drug addicts: ”

Nurses are like, “We understand [your situation], but we just get so many people who abuse the medication. We get a lot of drug addicts.” I’m like, “Yes, but that’s not everybody.” That isn’t everybody. I feel like as a chronic pain patient being responsible and being—I’ve been very much at times caught up in the crossfire of this opioid epidemic. (Irene, 40–49, British Columbia, treated with opioids)

Participants’ distinction between “us” (meaning people who live with chronic pain) and “them” (meaning people who are dependent on opioids or use them recreationally) used various arguments. Several participants claimed to be “one of a few” that would never become addicted to opioids:

In terms of addiction and habit, I’m one of the few people for whom it doesn’t cause … I don’t need my medication. When I don’t have any pain, I forget taking them, I don’t need them. (Allison, 50–59, Quebec, treated with opioids)

Not everyone gets addicted to these drugs. Until now, I didn’t take Tylenol 3 when I was working, I used to take it in the evening at home. I was able to function even if I didn’t use it for a few days. It was very light. I’ve been using it for four years, and now that I’m at home, I can use it all day, but my dosage hasn’t increased over time. So, I don’t think I would be the type of person who could develop problematic use of this medication. (Mireille, 40–49, Quebec, treated with opioids)

Several participants assumed that people who became dependent on opioid analgesics may have a predisposition for addiction. From their perspective, addiction was a problem with the person, not the substance:

From what I think, to get addicted, you need to like the effects. So, these are probably people who … most people who will use painkillers and become addicted are probably people who have already used [illicit] drugs, and who had a time in their life when they used it and they liked it. They may have rediscovered the euphoria they used to feel, which makes it easier for them to become addicted. (Jean-François, 60–69, Quebec, treated with opioids)

Indeed, many participants attempted to distinguish themselves from people who appreciate the effects of these drugs. They considered the opioid “high” as an adverse effect rather than a desirable effect. They emphasized that taking opioids had never been a choice for their pleasure but rather a necessity for pain relief. Some insisted that if they had a choice, they would prefer not using these drugs:

Some of the nurses think you’re drug-seeking, they think you’re coming there to get high, but I’m not getting high from this stuff. I don’t even like it when I don’t start to feel good from these drugs. […] I can see people are coming in [the ER] and wanting pain drugs, and I was seeing it while I was lying there. I don’t like them putting me in that category, that I’m drug-seeking, because I’m not drug-seeking. I’d rather not be on drugs. But I’m in a situation where I have no choice. (Patrick, 60–69, British Columbia, treated with opioids)

Distancing themselves from the devalued label of “addicts” could in turn lead some participants to stigmatize people who use illicit drugs. Some participants blamed those they called “addicts” for being responsible for the discriminations of patients with CNCP in the health care system:

I don’t know what’s happening, but there’s so many problems with the opioid crisis that those who actually need the medication are now having problems because of the bad apples that are creating the issues. We’re getting caught up and we’re getting the negative effects because of what’s happening from the overdoses and all of the reaction to it. (Irene, 40–49, British Columbia, treated with opioids)

However, for a minority of participants, the experience of discrimination fostered empathy with people who use illicit substances. After having experienced similar stigma in the health care system, some participants reported that they better understood these people’s difficulties and revised their previous negative judgments:

I think it takes time, it takes effort and it takes diligence to look into it and learn about it. And I even think of myself, like I lived [near a deprived neighborhood] in [date anonymized] and I thought completely differently about addicts and heroin users. I was frustrated by them because I lived in the area and they were everywhere, they were breaking into buildings. But I was also really out of touch with myself and my community and my world. And again, I kind of go back to the treatment that I went through, it’s allowed me to kind of open my eyes a little bit and open my mind to what’s going on and not just judge. (Samuel, 40–49, British Columbia, treated with opioids)

Sometimes, empathy for people who use illicit drugs and the will to distinguish themselves from the stigmatizing label of “addicts” were interlaced in participants’ narratives:

Because I was wrongly diagnosed as an addict, I’ve got a really strong connection with the whole addiction field, and I joined almost every addiction support group on the Internet. I got to know a lot of people in that community very, very, well. It started to become obvious to me that I wasn’t really one of them, I couldn’t relate to some of the behaviors at all, but I couldn’t judge them any longer. They became friends, and they loved me, and I loved them. Like, there are a lot of artists, musicians in that group of people, a lot of people who have had severe trauma, unimaginable. I’ve seen it from that angle. (Bettina, 70–79, British Columbia, treated with opioids)

I said to myself, “No, I don’t want to go to rehab.” It would have been another blow in my life. I have nothing against the people who go [to rehab], some people really need it, the people who are on the street and who use junk [illicit drugs], they have no choice. When they need it, they have to do it, but I didn’t want to. It would have been like another slap for me. I had enough! It’s not because I have something against these people. If they get there, it’s because they have a difficult life. I have no judgment on these people. (Annie, 60–69, Quebec, treated with opioids in the past)

## Discussion

This study highlights the collateral damage of the opioid overdose epidemic for the health care experiences of people who live with CNCP. We have shown how some patients can be deprived of their treatment due to recent opioid policy/practice changes and media coverage. We have also underscored how they may face discrimination in health care due to opioid-related stigma. Discrimination was most frequently reported with providers who were unfamiliar with participants’ medical history (especially in hospitals), and we identified structural inequities regarding opportunities to counter stigma. The opioid overdose epidemic also led some people with CNCP to distance themselves from the stigmatized identity of “addict.” This section provides several avenues for interpreting these findings and suggests opportunities for action.

Firstly, this study has shown that access to pain relief can be limited by recent guidelines promoting opioid tapering as a standardized medical response. Because recent literature has shown that the opioid overdose epidemic created many new challenges for physicians treating CNCP,^13^ it is crucial to offer pathways supporting clinical practice in this regard. Several of our participants felt that the specifics of their medical history were no longer considered by providers when making decisions about their opioid treatment. This result adds to the conclusions of one recent study highlighting patients’ loss of autonomy and control over their treatment since opioid policy changes.^8^ Many participants in our study also deplored that their personal needs for functioning and preferences for treatment were poorly acknowledged by providers. This may testify to a mismatch between patients’ and providers’ objectives^37,38^: Whereas most patients give importance to their daily quality of life, many providers may prioritize the avoidance of physical risks such as opioid dependence, with less consideration for pain and psychosocial dimensions. Our findings suggest that it would be valuable to find an intermediary path between both of these priorities. Opioid guidelines are essential to inform clinical practice, but they should be interpreted in the context of a comprehensive provider–patient interaction in which the patient’s preferences, feelings, and values are addressed. Conversations on pain should include the meaning of the pain experience and a patient’s goals in seeking care, which may help providers appreciate the complex and subjective nature of the pain experience.^39^ The development of shared decision making between providers and patients appears crucial to improve CNCP management in the context of the opioid overdose epidemic.^40^ Individualized and patient-centered approaches in medical decisions regarding opioid tapering should be prioritized,^41–43^ because some patients with CNCP may experience more barriers to ceasing opioids due to higher pain intensity or negative effects on mood, sleep, and quality of life.^44^ Indeed, standardized tapering that does not take into account each patient’s situation may lead to detrimental effects on both pain and psychological outcomes. Some research suggested that though long-term opioid therapy may reduce pain severity and improve quality of life for some patients,^45^ it remains difficult to identify a typical profile in terms of health or sociodemographic predictors of improvement.^46^ This should encourage development of biopsychosocial and patient-centered approaches to pain management, focusing on shared decisions and acknowledging the singularity of each patient. The use of clinical pain assessment tools^47,48^ and qualitative pain profiles^49,50^ can assist health care providers in conducting multidimensional pain assessments and foster collaborative provider–patient relationships.^51^

Secondly, this study has provided a deep understanding of discrimination and stigma that patients with CNCP can face in the health care system. Opioid-related stigma in the treatment of CNCP did not begin with the opioid overdose epidemic^23,24,52–54^; however, this epidemic has exacerbated stigma experiences in health care settings among individuals living with pain, by turning the “addict” identity into the key reference for qualifying people who use opioids.^14,15^ Though our study was conducted in two Canadian provinces that were diversely impacted by the opioid overdose epidemic, we did not identify salient differences between the experiences of participants from both provinces. Participants from Quebec reported stigma and barriers to care related to the opioid overdose epidemic, despite this epidemic being less prevalent in their province.

Stigma has been conceptualized as a multilevel phenomenon involving both systemic and interactional dimensions.^27,55,56^ Negative labels and stereotyping during interpersonal interactions can reinforce the systemic discrimination of stigmatized groups and negatively impact public health outcomes.^28,57^ Our study contributes to the understanding of how a macrolevel context (i.e., the opioid overdose epidemic) can convey stigma in micro interactions through prejudices and discrimination in health care. This can help improve clinical practice in the field of pain and beyond. We identified how and in what ways people with CNCP experience opioid-related stigma across the health care system and how stigma can impair access to pain management. Participants were particularly exposed to stigma with providers who were unfamiliar with their medical history. Emergency departments in particular and hospitals more broadly appeared in the participants’ narratives as being discriminatory places. Participants were exposed to both care denials and stigmatizing labels that could be affixed durably to their medical records. In a previous study among people who use illicit drugs and suffer from concomitant chronic pain, we have already shown that the “addict” label can lead to detrimental consequences for further access to health care services and pain management.^58,59^ Furthermore, experiencing discrimination can lead to impairments at the physical and psychological levels, as illustrated in a study showing that social discriminations can indirectly contribute to pain chronicity.^60^ Providers’ stigma regarding opioids can also create feelings of guilt and low self-esteem among people with CNCP treated with these medications.^23^ This supports the need to take actions to reduce stigma and improve access to respectful health care services for all patients treated with opioids, regardless of whether they live with pain alone or with concurrent substance use disorder. Harm reduction and equity-oriented approaches to opioid consumption^61^ are useful resources to help providers during patient encounters that they may find challenging or worrisome.

In our study, experiences of discrimination were almost absent with providers who knew their patients for a long time. Participants described some extremely positive relationships with trusted family physicians, specialists, pharmacists, and/or nurses who provided long-term follow-up for their pain. Unfortunately, such relationships were not available to all participants. In the context of ongoing shortages of primary care physicians, the absence of an abiding treating provider dramatically increased vulnerability and exposure to prejudices, stigma, and care denials. Priority actions to reduce discrimination in pain management should thus focus on improving access to treating family physicians, as well as educating and supporting all providers in the use of nonstigmatizing approaches to CNCP and opioids.

One emerging finding of this study is that participants had unequal resources to face discrimination in health care. The patients experiencing more issues in being listened to and believed were those who were unfamiliar with communication skills and scientific/medical literacy. Countering the “addict” label often requires knowledge and advocacy skills that many people with CNCP never had the chance to acquire. These participants’ experiences could be understood using the concept of “epistemic injustice” or, more precisely, “testimonial injustice,”^62,63^ which refers to sociocultural inequities in being listened to and believed and in accessing other peoples’ trust. To our knowledge, only one article used this concept to analyze the health care experiences of patients with chronic pain.^64^ This emerging finding in our study thus opens promising research avenues for further exploration of chronic pain management through the lens of epistemic injustice.

Our findings demonstrate the importance of fully including social determinants of health, such as sociodemographic characteristics and cultural health capital,^65^ in policies and clinical approaches to pain diagnosis and management. It complements research highlighting the negative impact of other social inequities such as racism and gender-based discriminations for pain management.^66,67^ It is crucial to develop tools that assist providers in better listening to CNCP patients independent of their opioid use, their social characteristics, or their ease with medical language. For example, using clinical training approaches focused on “structural competency” could help providers identify and reduce structural power relationships during clinical interactions.^68^ Patients’ access to information about their condition and health care services should also be improved.

Finally, one last important finding in this study is the distinction between “pain patients” and “drug addicts” that people with CNCP tend to use when referring to the opioid overdose epidemic. Distinction between “us” and “them” is a key characteristic of the stigmatization process.^27^ Some participants used stigmatizing language when talking about people who use illicit drugs. They typically felt distant from the world of illicit substance use and were upset that some providers could confuse them with those they called “addict.” Some patients’ strategies to avoid being negatively labeled in their everyday life were previously documented.^54,69^ Our study showed that the opioid overdose epidemic seems to have increased prejudice among patients with CNCP regarding people who are dependent on opioids or use them illicitly. This raises concerns in terms of cohesion and solidarity between people in pain, given that many people who use illicit drugs also suffer from chronic pain^70,71^ and the distinction between “patients” and “addicts” appears irrelevant in practice.^72,73^ Some recent research showed that contrasting “good” and “bad” opioid users is the norm in current media coverage and common representations of opioids.^14,15,74^ Future public information campaigns related to the opioid overdose epidemic should avoid this, given that it may reinforce prejudices among both patients with pain and the public. Another novel finding in this study is the case of some participants who reported increased empathy toward people who use illicit drugs. This suggests that promoting similarities rather than differences between opioid users may help change people’s views. Stigma should not be used as a public health strategy to address the opioid overdose epidemic.^26^ Destigmatizing all forms of opioid use appears as a win–win option for everyone, including people who live with CNCP and people who use illicit opioids.

### Strengths and Limitations

Despite chronic pain being a multidimensional phenomenon including social dimensions and nonmeasurable experiences,^39,75^ qualitative and social research still remains underdeveloped in this field. We believe that this study is an important contribution to foster the understanding of patients’ experiences and perspectives. The results obtained contribute to advancing scientific knowledge and clinical practice for the management of chronic pain in the context of the opioid overdose epidemic. Giving voice to people living with pain is crucial to improve health policies and promote nonstigmatizing approaches regarding pain and opioids. Furthermore, our study highlighted the role of structural inequities in the health care experiences of people living with CNCP. This represents a major knowledge improvement for the integration of social dimensions in multimodal approaches of pain management.

One limitation of this study may be due to purposive sampling. Participants who reported little or no impact of the opioid overdose epidemic on their situation were not included in this qualitative sample. However, we recruited participants with diversified characteristics to maximize validity of data.^35^ Participants reported a broad range of experiences including positive aspects (e.g., relationships of trust with providers), which gives nuance to negative impacts. Moreover, even though the problems raised in this article, such as discrimination in health care services, do not affect all people suffering from CNCP, addressing these issues remains of major importance given that the most vulnerable patients may be primarily affected.

## Conclusion

This study revealed the needs to improve access to health services for people living with CNCP and to address the issue of discrimination in health care for those who use opioid analgesics. Future health policies should promote nonstigmatizing clinical responses to people who use opioids. Health care settings and systems should provide staff with adequate resources and training to help them more effectively meet the needs of people in pain. Health policies and education programs should support providers in recognizing the importance of assessing and treating pain and increase their awareness of the negative impact of stigma on patient outcomes.
